# Supplementary material for: Little hope for the polyploid endemic Pyrenean Larkspur (Delphinium montanum): Evidences from population genomics and Ecological Niche Modeling
Source: Ecol Evol. 2022 Mar 18;12(3):e8711. doi: 10.1002/ece3.8711 (PMC8932081; doi:10.1002/ece3.8711)
Supplement: Supplementary file 1 — Appendix S1‐S4 [file ECE3-12-e8711-s001.pdf]

Supplementary Appendix S1

Individuals of *Delphinium montanum* sampled in this study. Each row represents an individual for which sampling locality, geographic coordinates and collector name(s) are reported. There are 106 individuals in total.

Individual ID	Locality	Longitude (°)	Latitude (°)	Elevation (m)	Collector
20-Dmo-001	Orri de Baix	2.122	42.446	2170	JB
20-Dmo-002	Orri de Baix	2.122	42.446	2171	JB
20-Dmo-003	Orri de Baix	2.122	42.446	2170	JB
20-Dmo-004	Orri de Baix	2.122	42.446	2171	JB
20-Dmo-005	Orri de Baix	2.122	42.446	2175	JB
20-Dmo-006	Orri de Baix	2.123	42.446	2171	JB
20-Dmo-007	Orri de Baix	2.114	42.444	2020	JB
20-Dmo-008	Orri de Baix	2.114	42.444	2017	JB
20-Dmo-009	Orri de Baix	2.115	42.443	2036	JB
20-Dmo-010	Orri de Baix	2.115	42.443	2053	JB
20-Dmo-011	Orri de Baix	2.117	42.442	2108	JB
20-Dmo-012	Orri de Baix	2.117	42.442	2117	JB
20-Dmo-013	Nohèdes	2.263	42.616	1755	JB
20-Dmo-014	Nohèdes	2.263	42.616	1758	JB
20-Dmo-015	Nohèdes	2.263	42.616	1760	JB
20-Dmo-016	Nohèdes	2.263	42.615	1760	JB
20-Dmo-017	Nohèdes	2.263	42.615	1761	JB
20-Dmo-018	Nohèdes	2.263	42.615	1759	JB
20-Dmo-019	Nohèdes	2.263	42.615	1758	JB
20-Dmo-020	Nohèdes	2.263	42.615	1759	JB
20-Dmo-021	Nohèdes	2.263	42.615	1760	JB
20-Dmo-022	Nohèdes	2.263	42.615	1758	JB
20-Dmo-023	Nohèdes	2.263	42.616	1757	JB
20-Dmo-024	Nohèdes	2.263	42.616	1753	JB
20-Dmo-025	Vallter	2.266	42.427	2145	AV
20-Dmo-026	Vallter	2.266	42.427	2145	AV
20-Dmo-027	Vallter	2.264	42.426	2140	AV
20-Dmo-028	Vallter	2.264	42.426	2140	AV
20-Dmo-029	Vallter	2.264	42.426	2140	AV
20-Dmo-030	Vallter	2.264	42.426	2140	AV
20-Dmo-031	Vallter	2.264	42.426	2140	AV
20-Dmo-032	Vallter	2.264	42.426	2140	AV
20-Dmo-033	Vallter	2.264	42.426	2140	AV
20-Dmo-034	Vallter	2.264	42.426	2140	AV
20-Dmo-035	Vallter	2.264	42.426	2140	AV
20-Dmo-036	Vallter	2.264	42.426	2140	AV
20-Dmo-037	Pedraforca	NA	NA	NA	PA, IA
20-Dmo-038	Pedraforca	1.701	42.238	NA	PA, IA
20-Dmo-039	Pedraforca	1.705	42.236	NA	PA, IA
20-Dmo-040	Serra Pedregosa	1.688	42.277	NA	PA, IA
20-Dmo-041	Cambre d'Aze	2.131	42.451	2723	JP, LS
20-Dmo-042	Cambre d'Aze	2.131	42.451	2720	JP, LS
20-Dmo-043	Cambre d'Aze	2.124	42.456	2405	JP, LS
20-Dmo-044	Cambre d'Aze	2.124	42.456	2314	JP, LS
20-Dmo-045	Serra Pedregosa	1.688	42.276	NA	PA, IA
20-Dmo-046	Tosa	1.886	42.315	NA	PA, IA
20-Dmo-047	Tosa	1.886	42.315	NA	PA, IA
20-Dmo-048	Batanist	1.688	42.291	NA	PA, IA
20-Dmo-049	Cambre d'Aze	2.130	42.450	2649	JP, LS
20-Dmo-050	Cambre d'Aze	2.130	42.450	2637	JP, LS

20-Dmo-051	Cambre d'Aze	2.130	42.450	2629	JP, LS
20-Dmo-052	Cambre d'Aze	2.130	42.450	2626	JP, LS
20-Dmo-053	Cambre d'Aze	2.129	42.449	2558	JP, LS
20-Dmo-054	Cambre d'Aze	2.129	42.449	2585	JP, LS
20-Dmo-055	Cambre d'Aze	2.130	42.449	2587	JP, LS
20-Dmo-056	Cambre d'Aze	2.130	42.449	2598	JP, LS
20-Dmo-057	Pedraforca	1.706	42.237	NA	JP, LS
20-Dmo-058	Pedraforca	1.705	42.236	NA	JP, LS
20-Dmo-059	Pedraforca	1.706	42.236	NA	JP, LS
20-Dmo-060	Pedraforca	1.706	42.236	NA	JP, LS
20-Dmo-061	Pedraforca	1.703	42.237	NA	JP, LS
20-Dmo-062	Pedraforca	1.705	42.238	NA	JP, LS
20-Dmo-065	Tosa	1.886	42.315	NA	PA, IA, QT
20-Dmo-066	Tosa	1.886	42.315	NA	PA, IA, QT
20-Dmo-067	Tosa	1.886	42.315	NA	PA, IA, QT
20-Dmo-068	Bastanist	1.688	42.289	NA	PA, IA
20-Dmo-069	Bastanist	1.688	42.289	NA	PA, IA
20-Dmo-070	Bastanist	1.688	42.289	NA	PA, IA
20-Dmo-071	Bastanist	1.688	42.289	NA	PA, IA
20-Dmo-072	Bastanist	1.688	42.289	NA	PA, IA
20-Dmo-073	Serra Pedregosa	1.689	42.278	NA	PA, IA
20-Dmo-074	Serra Pedregosa	1.687	42.278	NA	PA, IA
20-Dmo-075	Tosa	1.886	42.315	NA	PA, IA, QT
20-Dmo-076	Tosa	1.886	42.315	NA	PA, IA, QT
20-Dmo-077	Tosa	1.886	42.315	NA	PA, IA, QT
20-Dmo-078	Tosa	1.886	42.315	NA	PA, IA, QT
20-Dmo-079	Tosa	1.886	42.315	NA	PA, IA, QT
20-Dmo-080	Bastanist	1.688	42.290	NA	PA, IA
20-Dmo-081	Serra Pedregosa	1.687	42.278	NA	PA, IA
20-Dmo-082	Serra Pedregosa	1.687	42.278	NA	PA, IA
20-Dmo-083	Serra Pedregosa	1.687	42.278	NA	PA, IA
20-Dmo-084	Serra Pedregosa	1.687	42.278	NA	PA, IA
20-Dmo-085	Tosa	1.886	42.315	NA	PA, IA, QT
20-Dmo-086	Tosa	1.886	42.315	NA	PA, IA, QT
20-Dmo-087	Bastanist	1.688	42.289	NA	PA, IA
20-Dmo-088	Bastanist	1.688	42.290	NA	PA, IA
20-Dmo-089	Pedraforca	1.701	42.238	NA	PA, IA
20-Dmo-090	Serra Pedregosa	1.688	42.277	NA	PA, IA
20-Dmo-091	Serra Pedregosa	1.688	42.277	NA	PA, IA
20-Dmo-092	Serra Pedregosa	1.687	42.277	NA	PA, IA
20-Dmo-093	Serra Pedregosa	1.687	42.277	NA	PA, IA
20-Dmo-094	Bastanist	1.689	42.288	NA	PA, IA
20-Dmo-095	Bastanist	1.689	42.288	NA	PA, IA
20-Dmo-096	Bastanist	1.688	42.291	NA	PA, IA
20-Dmo-097	Torreta de Cadí	1.574	42.282	NA	PA, IA
20-Dmo-098	Torreta de Cadí	1.574	42.283	NA	PA, IA
20-Dmo-099	Torreta de Cadí	1.574	42.283	NA	PA, IA
20-Dmo-100	Torreta de Cadí	1.574	42.282	NA	PA, IA
20-Dmo-101	Torreta de Cadí	1.574	42.283	NA	PA, IA
20-Dmo-102	Torreta de Cadí	1.574	42.282	NA	PA, IA
20-Dmo-103	Torreta de Cadí	1.574	42.282	NA	PA, IA
20-Dmo-104	Torreta de Cadí	1.574	42.282	NA	PA, IA
20-Dmo-105	Torreta de Cadí	1.574	42.283	NA	PA, IA
20-Dmo-106	Torreta de Cadí	1.574	42.283	NA	PA, IA
20-Dmo-107	Torreta de Cadí	1.574	42.283	NA	PA, IA
20-Dmo-108	Torreta de Cadí	1.574	42.282	NA	PA, IA

Geographic coordinates are in WGS84.

Collectors' names abbreviations: JB (Joris Bertrand), PA (Pere Aymerich), AV (Albert Vila), JP (Josep Parrera), IA (Irene Abad), Lucie Schaad and QT (Queralt Tor)

Supplementary Appendix S2

Evolution of mean read depth depending on several combination of the key parameters of stacks (*M*, *n* and *m*) on a representative subset of 12 individuals. Individuals with minimum and maximum mean read depth are indicated in bold.

Individual	M1n1m3	M2n1m3	M2n2m3	M3n3m3	M4n4m3	M5n5m3	M6n6m3	M7n7m3	M8n8m3	M9n9m3
20-Dmo-001	27.93	29.42	29.42	30.51	31.41	32.25	32.94	33.47	33.9	34.25
20-Dmo-013	32.77	34.4	34.4	35.65	36.7	37.67	38.46	39.14	39.65	40.04
20-Dmo-025	35.86	37.56	37.55	38.83	39.84	40.78	41.55	41.14	42.61	43
20-Dmo-037	38.55	40.52	40.53	41.95	43.08	44.1	44.95	45.67	46.2	46.61
20-Dmo-040	34.5	36.53	36.53	37.93	38.97	39.91	40.74	41.38	41.9	42.35
20-Dmo-041	34.59	36.19	36.19	37.35	38.46	39.37	40.17	40.79	41.3	41.67
20-Dmo-046	30.29	31.84	31.84	32.96	33.81	34.56	35.24	35.77	36.18	36.55
20-Dmo-050	29.15	30.78	30.78	31.94	32.93	33.79	34.59	35.17	35.62	35.94
20-Dmo-059	25.36	26.75	26.75	27.83	28.73	29.53	30.21	30.81	31.25	31.6
20-Dmo-068	35.19	37.19	37.19	38.53	39.62	40.54	41.36	42.01	42.52	42.92
20-Dmo-097	26.43	27.78	27.78	28.79	29.73	30.54	31.27	31.84	32.25	32.63
20-Dmo-105	49.54	52.23	52.23	53.89	55.23	56.37	57.25	57.95	58.55	58.95

-*m*: minimum number of identical raw reads required to form a putative allele
-*M*: number of mismatches allowed between alleles to form a locus
-*n*: number of mismatches allowed between loci during construction of the catalog

Optimization of several key parameters of stacks (*M*, *n* and *m*) on a representative subset of 12 individuals. The number of total loci, removed loci, kept loci, sites filtered sites, SNPs, assembled loci, polymorphic loci and new polymorphic loci compared to the previous combination of parameters are indicated. Data were filtered with the *populations* script from STACKS following the '*80% rule*' (*i.e.* keeping only loci shared by at least 80% of samples). The selected combination is indicated in bold.

**Parameters**			**Total loci**	**Removed loci**	**Kept loci**	**Sites**	**Filtered sites**	**SNPs**	**Assembled loci**	**Polymorphic loci**	**New Polymorphic loci**
**M**	**n**	**m**									
1	1	3	91451	71781	19670	6692290	1455	16697	19669	10798	NA
2	1	3	87320	68155	19165	6530249	2555	19330	19165	11220	422
2	2	3	84607	64906	19701	6703995	2768	20496	19701	11670	450
3	3	3	81420	62332	19088	6498001	4452	22911	19088	11825	155
4	4	3	79107	60650	18457	6288499	6817	24776	18457	11825	0
5	5	3	77437	59628	17809	6075321	9073	26667	17809	11711	-114
6	6	3	76078	58776	17302	5904844	11147	28488	17302	11649	-62
7	7	3	74975	58063	16912	5778683	13033	29671	16912	11579	-70
8	8	3	74289	57621	16668	5698383	14692	31317	16667	11563	-16
9	9	3	73421	57029	16392	5610276	16184	32093	16391	11489	-74

Supplementary Appendix S3

Pairwise genetic differentiation (G_{ST} values; Nei, 1987, G'_{ST}; Hendrick, 2005, G''_{ST}; Meirmans & Hedrick, 2011 and Jost's *D*) and pairwise geographic distances (in km). All values were found to be statistically significant ($p < 0.05$).

G_{ST}/Distance	Orri de Baix	Nohèdes	Vallter	Pedraforca	Serra Pedregosa	Cambre d'Aze	Tosa	Bastanist	Torreta de Cadi
Orri de Baix		0.441	0.433	0.394	0.390	0.156	0.427	0.382	0.483
Nohèdes	22		0.480	0.439	0.437	0.404	0.493	0.436	0.527
Vallter	12	21		0.426	0.438	0.401	0.490	0.426	0.518
Pedraforca	41	62	51		0.264	0.357	0.383	0.264	0.360
Serra Pedregosa	40	60	50	5		0.360	0.380	0.153	0.376
Cambre d'Aze	1	21	11	42	41		0.394	0.353	0.449
Tosa	24	45	33	17	17	25		0.375	0.480
Bastanist	39	59	50	6	1	40	17		0.369
Torreta de Cadi	48	68	59	12	9	49	26	9	

G'_{ST}/Distance	Orri de Baix	Nohèdes	Vallter	Pedraforca	Serra Pedregosa	Cambre d'Aze	Tosa	Bastanist	Torreta de Cadi
Orri de Baix		0.452	0.443	0.404	0.400	0.172	0.436	0.393	0.493
Nohèdes	22		0.490	0.447	0.448	0.416	0.503	0.447	0.537
Vallter	12	21		0.434	0.448	0.412	0.499	0.437	0.527
Pedraforca	41	62	51		0.280	0.371	0.393	0.279	0.372
Serra Pedregosa	40	60	50	5		0.372	0.391	0.167	0.387
Cambre d'Aze	1	21	11	42	41		0.405	0.365	0.46
Tosa	24	45	33	17	17	25		0.386	0.491
Bastanist	39	59	50	6	1	40	17		0.38

Torreta de Cadi	48	68	59	12	9	49	26	9	

G''_{ST}/ Jost's *D*	Orri de Baix	Nohèdes	Vallter	Pedraforca	Serra Pedregosa	Cambre d'Aze	Tosa	Bastanist	Torreta de Cadi
Orri de Baix		0.542	0.531	0.514	0.507	0.214	0.533	0.499	0.606
Nohèdes	0.163		0.579	0.559	0.559	0.510	0.604	0.559	0.650
Vallter	0.158	0.172		0.543	0.559	0.505	0.600	0.546	0.639
Pedraforca	0.185	0.202	0.193		0.372	0.484	0.501	0.372	0.479
Serra Pedregosa	0.178	0.199	0.200	0.127		0.483	0.498	0.221	0.496
Cambre d'Aze	0.051	0.159	0.158	0.180	0.177		0.506	0.475	0.579
Tosa	0.171	0.202	0.201	0.179	0.174	0.169		0.492	0.605
Bastanist	0.175	0.201	0.194	0.128	0.065	0.174	0.172		0.489
Torreta de Cadi	0.223	0.243	0.235	0.169	0.176	0.220	0.224	0.174	

Supplementary Appendix S4

Summary of the 135 models of Maxent ENMs generated by ENMeval. The two best models are indicated in bold.

	settings	features	rm	train.AUC	avg.test.AUC	var.test.AUC	avg.diff.AUC	var.diff.AUC	avg.test.orMTP	var.test.orMTP	avg.test.or10pct	var.test.or10pct	AICc	delta.AICc	w.AIC	parameters	sel.cri	rankAIC
**1**	L_0.5	L	0.5	0.992	0.984	0.000	0.008	0.001	0.250	0.250	0.375	0.229	164.270	12.016	0.001	4		18
**2**	P_0.5	P	0.5	0.992	0.984	0.000	0.006	0.000	0.250	0.083	0.375	0.229	179.791	27.537	0.000	5		96
**3**	Q_0.5	Q	0.5	0.993	0.986	0.000	0.007	0.000	0.250	0.250	0.375	0.229	161.481	9.228	0.003	4		14
**4**	H_0.5	H	0.5	0.997	0.988	0.000	0.006	0.000	0.250	0.250	0.375	0.229	NA	NA	NA	9		127
**5**	LP_0.5	LP	0.5	0.993	0.984	0.000	0.007	0.000	0.250	0.250	0.375	0.229	175.714	23.461	0.000	5		77
**6**	LQ_0.5	LQ	0.5	0.994	0.988	0.000	0.006	0.000	0.250	0.250	0.375	0.229	158.292	6.038	0.016	4		7
**7**	LH_0.5	LH	0.5	0.997	0.986	0.000	0.006	0.000	0.250	0.250	0.375	0.229	NA	NA	NA	9		128
**8**	PQ_0.5	PQ	0.5	0.994	0.988	0.000	0.006	0.000	0.250	0.250	0.375	0.229	158.834	6.580	0.012	4		9
**9**	PH_0.5	PH	0.5	0.997	0.988	0.000	0.006	0.000	0.250	0.250	0.375	0.229	NA	NA	NA	10		129
**10**	QH_0.5	QH	0.5	0.997	0.987	0.000	0.006	0.000	0.250	0.250	0.375	0.229	NA	NA	NA	8		130
**11**	LPQ_0.5	LPQ	0.5	0.994	0.987	0.000	0.006	0.000	0.250	0.250	0.375	0.229	158.834	6.581	0.012	4		10
**12**	LPH_0.5	LPH	0.5	0.997	0.988	0.000	0.006	0.000	0.250	0.250	0.375	0.229	NA	NA	NA	10		131
**13**	LQH_0.5	LQH	0.5	0.997	0.987	0.000	0.006	0.000	0.250	0.250	0.375	0.229	NA	NA	NA	8		132
**14**	PQH_0.5	PQH	0.5	0.997	0.988	0.000	0.007	0.000	0.250	0.250	0.375	0.229	NA	NA	NA	9		133
**15**	LPQH_0.5	LPQH	0.5	0.997	0.988	0.000	0.007	0.000	0.250	0.250	0.375	0.229	NA	NA	NA	10		134
**16**	L_1	L	1	0.992	0.982	0.000	0.008	0.000	0.250	0.250	0.375	0.229	167.297	15.043	0.000	4		32
**17**	P_1	P	1	0.989	0.979	0.000	0.007	0.000	0.250	0.083	0.333	0.056	190.123	37.869	0.000	5		110
**18**	Q_1	Q	1	0.993	0.985	0.000	0.007	0.000	0.375	0.229	0.375	0.229	163.697	11.443	0.001	4		17
**19**	H_1	H	1	0.996	0.987	0.000	0.005	0.000	0.250	0.250	0.250	0.250	262.110	109.857	0.000	7		125
**20**	LP_1	LP	1	0.990	0.980	0.000	0.008	0.000	0.375	0.229	0.542	0.174	186.641	34.388	0.000	5		106
**21**	LQ_1	LQ	1	0.994	0.987	0.000	0.006	0.000	0.250	0.250	0.375	0.229	160.280	8.026	0.006	4		11
**22**	LH_1	LH	1	0.995	0.985	0.000	0.006	0.000	0.250	0.250	0.250	0.250	NA	NA	NA	8		135
**23**	PQ_1	PQ	1	0.993	0.980	0.000	0.008	0.000	0.375	0.229	0.542	0.174	166.411	14.157	0.000	4		28

**24**	PH_1	PH		1	0.995	0.988	0.000	0.006	0.000	0.250	0.250	0.375	0.229	262.704	110.451	0.000	7	126
**25**	QH_1	QH		1	0.995	0.986	0.000	0.005	0.000	0.250	0.250	0.375	0.229	165.298	13.044	0.000	5	27
**26**	LPQ_1	LPQ		1	0.993	0.980	0.000	0.008	0.000	0.375	0.229	0.542	0.174	166.411	14.157	0.000	4	29
**27**	LPH_1	LPH		1	0.995	0.987	0.000	0.006	0.000	0.250	0.250	0.375	0.229	190.731	38.477	0.000	6	114
**28**	LQH_1	LQH		1	0.995	0.986	0.000	0.005	0.000	0.250	0.250	0.375	0.229	165.294	13.041	0.000	5	26
**29**	PQH_1	PQH		1	0.995	0.987	0.000	0.006	0.000	0.250	0.250	0.375	0.229	190.594	38.341	0.000	6	111
**30**	LPQH_1	LPQH		1	0.995	0.987	0.000	0.006	0.000	0.250	0.250	0.375	0.229	190.594	38.341	0.000	6	112
**31**	L_1.5	L	1.5		0.991	0.978	0.000	0.009	0.000	0.375	0.229	0.625	0.229	171.977	19.724	0.000	4	55
**32**	P_1.5	P	1.5		0.982	0.978	0.000	0.008	0.000	0.250	0.083	0.417	0.083	180.445	28.191	0.000	3	99
**33**	Q_1.5	Q	1.5		0.992	0.981	0.000	0.008	0.000	0.375	0.229	0.542	0.174	167.003	14.749	0.000	4	30
**34**	H_1.5	H	1.5		0.995	0.986	0.000	0.006	0.000	0.250	0.250	0.250	0.250	195.376	43.123	0.000	6	122
**35**	LP_1.5	LP	1.5		0.984	0.978	0.000	0.008	0.000	0.250	0.083	0.417	0.083	174.697	22.443	0.000	3	72
**36**	LQ_1.5	LQ	1.5		0.994	0.984	0.000	0.007	0.000	0.375	0.229	0.375	0.229	163.206	10.952	0.001	4	15
**37**	LH_1.5	LH	1.5		0.995	0.984	0.000	0.007	0.000	0.250	0.250	0.375	0.229	194.601	42.347	0.000	6	121
**38**	PQ_1.5	PQ	1.5		0.986	0.979	0.000	0.009	0.000	0.375	0.229	0.625	0.229	178.614	26.360	0.000	4	92
**39**	PH_1.5	PH	1.5		0.995	0.988	0.000	0.005	0.000	0.250	0.250	0.375	0.229	158.595	6.341	0.014	4	8
**40**	QH_1.5	QH	1.5		0.995	0.987	0.000	0.005	0.000	0.250	0.250	0.375	0.229	193.233	40.980	0.000	6	118
**41**	LPQ_1.5	LPQ	1.5		0.986	0.979	0.000	0.009	0.000	0.375	0.229	0.542	0.174	178.614	26.360	0.000	4	93
**42**	LPH_1.5	LPH	1.5		0.995	0.986	0.000	0.006	0.000	0.250	0.250	0.375	0.229	171.530	19.276	0.000	5	52
**43**	LQH_1.5	LQH	1.5		0.995	0.987	0.000	0.005	0.000	0.250	0.250	0.375	0.229	193.233	40.980	0.000	6	119
**44**	PQH_1.5	PQH	1.5		0.994	0.986	0.000	0.006	0.000	0.250	0.250	0.500	0.167	195.671	43.417	0.000	6	123
**45**	LPQH_1.5	LPQH	1.5		0.994	0.985	0.000	0.006	0.000	0.250	0.250	0.500	0.167	195.671	43.417	0.000	6	124
**46**	L_2	L		2	0.987	0.976	0.000	0.010	0.000	0.375	0.229	0.625	0.229	178.226	25.972	0.000	4	91
**47**	P_2	P		2	0.982	0.977	0.000	0.009	0.000	0.125	0.063	0.500	0.167	181.826	29.572	0.000	3	100
**48**	Q_2	Q		2	0.992	0.980	0.000	0.009	0.000	0.375	0.229	0.625	0.229	171.343	19.089	0.000	4	51
**49**	H_2	H		2	0.994	0.982	0.000	0.008	0.000	0.250	0.250	0.375	0.229	177.171	24.917	0.000	5	85
**50**	LP_2	LP		2	0.982	0.978	0.000	0.007	0.000	0.250	0.083	0.500	0.167	172.358	20.105	0.000	2	59
**51**	LQ_2	LQ		2	0.993	0.981	0.000	0.008	0.000	0.375	0.229	0.625	0.229	167.187	14.933	0.000	4	31

**52**	LH_2	LH		2	0.994	0.984	0.000	0.007	0.000	0.250	0.250	0.375	0.229	163.386	11.132	0.001	4		16
**53**	PQ_2	PQ		2	0.983	0.979	0.000	0.007	0.000	0.250	0.083	0.500	0.167	170.000	17.746	0.000	2		46
**54**	**PH_2**	**PH**		**2**	**0.995**	**0.989**	**0.000**	**0.005**	**0.000**	**0.250**	**0.250**	**0.375**	**0.229**	**174.628**	**22.374**	**0.000**	**5**	**AUCmtp. AUC10**	**71**
**55**	**QH_2**	**QH**		**2**	**0.994**	**0.988**	**0.000**	**0.005**	**0.000**	**0.250**	**0.250**	**0.375**	**0.229**	**152.254**	**0.000**	**0.327**	**3**	**LowAIC**	**1**
**56**	LPQ_2	LPQ		2	0.983	0.979	0.000	0.008	0.000	0.250	0.083	0.500	0.167	170.000	17.746	0.000	2		45
**57**	LPH_2	LPH		2	0.994	0.984	0.000	0.008	0.000	0.125	0.063	0.375	0.229	176.919	24.665	0.000	5		84
**58**	LQH_2	LQH		2	0.994	0.988	0.000	0.005	0.000	0.250	0.250	0.375	0.229	152.254	0.000	0.327	3		2
**59**	PQH_2	PQH		2	0.993	0.985	0.000	0.006	0.000	0.125	0.063	0.375	0.229	164.505	12.252	0.001	4		21
**60**	LPQH_2	LPQH		2	0.993	0.985	0.000	0.007	0.000	0.250	0.250	0.375	0.229	164.505	12.252	0.001	4		22
**61**	L_2.5	L	2.5		0.981	0.973	0.000	0.011	0.000	0.375	0.229	0.625	0.229	172.343	20.089	0.000	2		58
**62**	P_2.5	P	2.5		0.982	0.974	0.000	0.009	0.001	0.125	0.063	0.375	0.229	183.538	31.284	0.000	3		101
**63**	Q_2.5	Q	2.5		0.989	0.978	0.000	0.009	0.000	0.375	0.229	0.625	0.229	168.380	16.127	0.000	3		36
**64**	H_2.5	H	2.5		0.993	0.975	0.000	0.011	0.000	0.250	0.250	0.625	0.229	172.408	20.154	0.000	4		60
**65**	LP_2.5	LP	2.5		0.982	0.978	0.000	0.006	0.000	0.250	0.083	0.500	0.167	173.475	21.222	0.000	2		66
**66**	LQ_2.5	LQ	2.5		0.992	0.978	0.000	0.010	0.000	0.375	0.229	0.625	0.229	164.945	12.691	0.001	3		25
**67**	LH_2.5	LH	2.5		0.993	0.980	0.000	0.009	0.000	0.250	0.250	0.375	0.229	179.937	27.683	0.000	5		98
**68**	PQ_2.5	PQ	2.5		0.982	0.978	0.000	0.006	0.000	0.125	0.063	0.500	0.167	171.077	18.823	0.000	2		49
**69**	PH_2.5	PH	2.5		0.995	0.985	0.000	0.007	0.000	0.250	0.250	0.458	0.174	179.824	27.571	0.000	5		97
**70**	QH_2.5	QH	2.5		0.994	0.987	0.000	0.005	0.000	0.250	0.250	0.375	0.229	154.566	2.313	0.103	3		3
**71**	LPQ_2.5	LPQ	2.5		0.982	0.978	0.000	0.007	0.000	0.250	0.083	0.500	0.167	171.077	18.823	0.000	2		50
**72**	LPH_2.5	LPH	2.5		0.992	0.980	0.000	0.009	0.000	0.125	0.063	0.542	0.174	171.909	19.655	0.000	4		54
**73**	LQH_2.5	LQH	2.5		0.994	0.987	0.000	0.005	0.000	0.250	0.250	0.375	0.229	154.566	2.313	0.103	3		4
**74**	PQH_2.5	PQH	2.5		0.993	0.982	0.000	0.008	0.000	0.125	0.063	0.375	0.229	168.297	16.043	0.000	4		34
**75**	LPQH_2.5	LPQH	2.5		0.993	0.982	0.000	0.008	0.000	0.125	0.063	0.375	0.229	168.297	16.043	0.000	4		35
**76**	L_3	L		3	0.978	0.972	0.000	0.010	0.000	0.250	0.083	0.625	0.229	174.761	22.507	0.000	2		74
**77**	P_3	P		3	0.981	0.972	0.001	0.010	0.001	0.125	0.063	0.375	0.229	185.564	33.310	0.000	3		104
**78**	Q_3	Q		3	0.986	0.976	0.000	0.009	0.000	0.375	0.229	0.625	0.229	167.555	15.301	0.000	2		33

**79**	H_3	H		3	0.990	0.973	0.000	0.011	0.000	0.250	0.083	0.625	0.229	173.426	21.173	0.000	3	65
**80**	LP_3	LP		3	0.981	0.978	0.000	0.004	0.000	0.125	0.063	0.500	0.167	174.759	22.506	0.000	2	73
**81**	LQ_3	LQ		3	0.988	0.976	0.000	0.010	0.000	0.375	0.229	0.625	0.229	169.816	17.562	0.000	3	44
**82**	LH_3	LH		3	0.991	0.976	0.000	0.010	0.000	0.125	0.063	0.625	0.229	173.834	21.580	0.000	4	69
**83**	PQ_3	PQ		3	0.982	0.978	0.000	0.004	0.000	0.125	0.063	0.500	0.167	172.308	20.054	0.000	2	56
**84**	PH_3	PH		3	0.994	0.981	0.000	0.009	0.000	0.375	0.229	0.625	0.229	185.433	33.179	0.000	5	103
**85**	QH_3	QH		3	0.994	0.982	0.000	0.007	0.000	0.250	0.250	0.375	0.229	157.298	5.044	0.026	3	5
**86**	LPQ_3	LPQ		3	0.982	0.977	0.000	0.005	0.000	0.125	0.063	0.500	0.167	172.308	20.054	0.000	2	57
**87**	LPH_3	LPH		3	0.991	0.978	0.000	0.009	0.000	0.250	0.083	0.625	0.229	169.434	17.181	0.000	3	43
**88**	LQH_3	LQH		3	0.994	0.982	0.000	0.007	0.000	0.250	0.250	0.375	0.229	157.298	5.044	0.026	3	6
**89**	PQH_3	PQH		3	0.992	0.978	0.000	0.009	0.000	0.125	0.063	0.625	0.229	164.851	12.597	0.001	3	23
**90**	LPQH_3	LPQH		3	0.992	0.978	0.000	0.009	0.000	0.125	0.063	0.625	0.229	164.851	12.597	0.001	3	24
**91**	L_3.5	L	3.5		0.975	0.971	0.000	0.008	0.000	0.250	0.083	0.500	0.167	177.536	25.282	0.000	2	86
**92**	P_3.5	P	3.5		0.981	0.972	0.001	0.011	0.001	0.125	0.063	0.375	0.229	187.963	35.709	0.000	3	108
**93**	Q_3.5	Q	3.5		0.984	0.975	0.000	0.008	0.000	0.250	0.083	0.625	0.229	169.091	16.838	0.000	2	41
**94**	H_3.5	H	3.5		0.984	0.972	0.000	0.010	0.000	0.250	0.083	0.500	0.167	179.241	26.987	0.000	3	94
**95**	LP_3.5	LP	3.5		0.980	0.976	0.000	0.004	0.000	0.125	0.063	0.375	0.229	176.181	23.928	0.000	2	79
**96**	LQ_3.5	LQ	3.5		0.984	0.974	0.000	0.010	0.000	0.375	0.229	0.625	0.229	169.091	16.838	0.000	2	42
**97**	LH_3.5	LH	3.5		0.988	0.970	0.000	0.012	0.000	0.250	0.083	0.625	0.229	171.776	19.523	0.000	3	53
**98**	PQ_3.5	PQ	3.5		0.980	0.977	0.000	0.004	0.000	0.125	0.063	0.375	0.229	173.677	21.424	0.000	2	68
**99**	PH_3.5	PH	3.5		0.992	0.978	0.000	0.009	0.000	0.250	0.083	0.500	0.167	192.068	39.815	0.000	5	116
**100**	QH_3.5	QH	3.5		0.994	0.979	0.000	0.008	0.000	0.125	0.063	0.625	0.229	160.494	8.240	0.005	3	12
**101**	LPQ_3.5	LPQ	3.5		0.980	0.977	0.000	0.004	0.000	0.125	0.063	0.375	0.229	173.662	21.409	0.000	2	67
**102**	LPH_3.5	LPH	3.5		0.989	0.976	0.000	0.009	0.000	0.250	0.083	0.500	0.167	173.840	21.587	0.000	3	70
**103**	LQH_3.5	LQH	3.5		0.994	0.979	0.000	0.008	0.000	0.250	0.250	0.625	0.229	160.494	8.240	0.005	3	13
**104**	PQH_3.5	PQH	3.5		0.991	0.977	0.000	0.009	0.000	0.250	0.083	0.625	0.229	168.744	16.490	0.000	3	40
**105**	LPQH_3.5	LPQH	3.5		0.991	0.977	0.000	0.009	0.000	0.250	0.083	0.625	0.229	168.731	16.477	0.000	3	39
**106**	L_4	L		4	0.972	0.971	0.000	0.006	0.000	0.125	0.063	0.500	0.167	176.564	24.311	0.000	1	80

**107**	P_4	P		4	0.980	0.971	0.001	0.012	0.001	0.125	0.063	0.375	0.229	190.705	38.452	0.000	3	113
**108**	Q_4	Q		4	0.982	0.973	0.000	0.007	0.000	0.125	0.063	0.500	0.167	170.783	18.530	0.000	2	48
**109**	H_4	H		4	0.976	0.973	0.000	0.006	0.000	0.125	0.063	0.500	0.167	192.826	40.572	0.000	4	117
**110**	LP_4	LP		4	0.979	0.975	0.000	0.005	0.000	0.125	0.063	0.375	0.229	177.713	25.460	0.000	2	88
**111**	LQ_4	LQ		4	0.982	0.972	0.000	0.010	0.000	0.250	0.083	0.625	0.229	170.783	18.530	0.000	2	47
**112**	LH_4	LH		4	0.983	0.971	0.000	0.011	0.000	0.250	0.083	0.625	0.229	177.574	25.320	0.000	3	87
**113**	PQ_4	PQ		4	0.979	0.975	0.000	0.005	0.000	0.125	0.063	0.375	0.229	175.108	22.854	0.000	2	75
**114**	PH_4	PH		4	0.986	0.977	0.000	0.006	0.000	0.125	0.063	0.500	0.167	186.698	34.444	0.000	4	107
**115**	QH_4	QH		4	0.993	0.974	0.000	0.010	0.000	0.250	0.083	0.625	0.229	164.473	12.219	0.001	3	19
**116**	LPQ_4	LPQ		4	0.979	0.975	0.000	0.005	0.000	0.125	0.063	0.375	0.229	175.108	22.854	0.000	2	76
**117**	LPH_4	LPH		4	0.984	0.974	0.000	0.009	0.000	0.250	0.083	0.500	0.167	185.913	33.660	0.000	4	105
**118**	LQH_4	LQH		4	0.993	0.973	0.000	0.011	0.000	0.250	0.083	0.625	0.229	164.473	12.219	0.001	3	20
**119**	PQH_4	PQH		4	0.989	0.974	0.000	0.010	0.000	0.250	0.083	0.500	0.167	173.046	20.792	0.000	3	63
**120**	LPQH_4	LPQH		4	0.989	0.974	0.000	0.010	0.000	0.250	0.083	0.500	0.167	173.046	20.792	0.000	3	64
**121**	L_4.5	L	4.5		0.972	0.971	0.000	0.006	0.000	0.125	0.063	0.500	0.167	176.854	24.600	0.000	1	83
**122**	P_4.5	P	4.5		0.980	0.969	0.001	0.012	0.001	0.125	0.063	0.375	0.229	193.856	41.602	0.000	3	120
**123**	Q_4.5	Q	4.5		0.979	0.972	0.000	0.006	0.000	0.125	0.063	0.500	0.167	172.617	20.363	0.000	2	61
**124**	H_4.5	H	4.5		0.972	0.973	0.000	0.005	0.000	0.125	0.063	0.375	0.229	188.578	36.324	0.000	3	109
**125**	LP_4.5	LP	4.5		0.977	0.973	0.000	0.005	0.000	0.125	0.063	0.375	0.229	179.329	27.075	0.000	2	95
**126**	LQ_4.5	LQ	4.5		0.979	0.972	0.000	0.007	0.000	0.125	0.063	0.500	0.167	172.617	20.363	0.000	2	62
**127**	LH_4.5	LH	4.5		0.980	0.970	0.000	0.010	0.000	0.250	0.083	0.500	0.167	175.906	23.652	0.000	2	78
**128**	PQ_4.5	PQ	4.5		0.978	0.974	0.000	0.005	0.000	0.125	0.063	0.375	0.229	176.614	24.360	0.000	2	82
**129**	PH_4.5	PH	4.5		0.979	0.975	0.000	0.005	0.000	0.125	0.063	0.375	0.229	192.051	39.797	0.000	4	115
**130**	QH_4.5	QH	4.5		0.991	0.972	0.000	0.011	0.000	0.250	0.083	0.625	0.229	168.604	16.350	0.000	3	37
**131**	LPQ_4.5	LPQ	4.5		0.978	0.974	0.000	0.005	0.000	0.125	0.063	0.375	0.229	176.614	24.360	0.000	2	81
**132**	LPH_4.5	LPH	4.5		0.978	0.973	0.000	0.007	0.000	0.250	0.083	0.500	0.167	183.634	31.380	0.000	3	102
**133**	LQH_4.5	LQH	4.5		0.991	0.971	0.000	0.011	0.000	0.250	0.083	0.625	0.229	168.604	16.350	0.000	3	38
**134**	PQH_4.5	PQH	4.5		0.984	0.974	0.000	0.008	0.000	0.250	0.083	0.500	0.167	177.741	25.487	0.000	3	89

**135**	LPQH_4.5	LPQH	4.5	0.984	0.973	0.000	0.009	0.000	0.250	0.083	0.500	0.167	177.741	25.487	0.000	3	90
